# Precision Medicine and Artificial Intelligence: A Pilot Study on Deep Learning for Hypoglycemic Events Detection based on ECG

**DOI:** 10.1038/s41598-019-56927-5

**Published:** 2020-01-13

**Authors:** Mihaela Porumb, Saverio Stranges, Antonio Pescapè, Leandro Pecchia

**Affiliations:** 1 School of Engineering, University of Warwick, Coventry, CV4 7AL UK; 2 Department of Epidemiology and Biostatistics, Schulich School of Medicine & Dentistry, Western University, Ontario, Canada; 3 Department of Family Medicine, Schulich School of Medicine & Dentistry, Western University, Ontario, Canada; 4 Department of Population Health, Luxembourg Institute of Health, Luxembourg, Luxembourg; 5 Department of Electrical Engineering, University of Napoli “Federico II”, Naples, Italy

**Keywords:** Biomedical engineering, Computer science, Pre-diabetes

## Abstract

Tracking the fluctuations in blood glucose levels is important for healthy subjects and crucial diabetic patients. Tight glucose monitoring reduces the risk of hypoglycemia, which can result in a series of complications, especially in diabetic patients, such as confusion, irritability, seizure and can even be fatal in specific conditions. Hypoglycemia affects the electrophysiology of the heart. However, due to strong inter-subject heterogeneity, previous studies based on a cohort of subjects failed to deploy electrocardiogram (ECG)-based hypoglycemic detection systems reliably. The current study used personalised medicine approach and Artificial Intelligence (AI) to automatically detect nocturnal hypoglycemia using a few heartbeats of raw ECG signal recorded with non-invasive, wearable devices, in healthy individuals, monitored 24 hours for 14 consecutive days. Additionally, we present a visualisation method enabling clinicians to visualise which part of the ECG signal (e.g., T-wave, ST-interval) is significantly associated with the hypoglycemic event in each subject, overcoming the intelligibility problem of deep-learning methods. These results advance the feasibility of a real-time, non-invasive hypoglycemia alarming system using short excerpts of ECG signal.

## Introduction

There is considerable heterogeneity among different patients or patient groups that trials based on patients cohorts may fail to capture, resulting in inaccurate conclusions about the effectiveness of diagnostics or interventions in individuals^1–4^. Some drugs have been reported to be effective only in 1 out of 24 patients because of these inaccuracies^4^. This has triggered unprecedented interest in personalised medicine (or precision medicine), for informing the design of more effective diagnostics and treatments based on high-quality evidence gathered from individuals, rather than cohorts, considering their personal history, genome, environment, lifestyle, physiology and behaviours. Discussion on how to overcome existing barriers for designing proper precision medicine studies is still going on, and overcoming current limitations of traditional trials requires different strategies for therapeutic^4^ and diagnostics^5^, drugs^6,7^ and medical devices^8^.

For the latter, moving towards the paradigm of precision medicine can be even more critical. Medical devices effectiveness is dependent upon several factors including the environment in which are utilised (e.g., sterilised or not, temperature, humidity, dust), the experience of the operators, maintaining, servicing and preparing the medical device^9^. For instance, recent studies demonstrated that even the positioning of applied parts (e.g., probes, sensors) in a trial might affect the significance of the results^10^. Therefore, pursuing a precision medicine approach in trialling deep learning (DL)-based medical technologies requires particular attention in the way the study is designed, executed and its results analysed^11–13^.

The study presented in this paper aims at exploring the efficacy of a personalised DL-based system for the automatic detection of lower levels of glucose in real-life settings. To the best of authors’ knowledge, this is a novel approach providing promising results, overcoming the limitations of previous attempts not based on personalised approaches and not employing DL methods.

Tracking the fluctuations in the blood glucose level is relevant for both healthy individuals and diabetic patients. High glucose levels (hyperglycemia) result in long-term complications and can damage the kidneys, nerves, blood vessels in the eye and can bring many other complications^14^. Low blood glucose levels (hypoglycemia) may result in acute short-term alterations of health status such as confusion, irritability, palpitations, feeling shaky and sweaty and can even result in severe loss of attention, coma or death^15^. In fact, hypoglycemia can be particularly dangerous during specific activities requiring great attention (e.g. while driving, performing complicated surgeries). Thus, technologies for non-invasive, continuous monitoring of glucose concentration aiming at early-detecting hypoglycemic events are highly required.

The most diffuse methods for blood glucose testing consist of analysing a drop of blood resulted from a finger prick. However, this method does not allow continuous monitoring, is invasive, cumbersome, expensive, and it has been demonstrated that it affects patient compliance with the glucose measurements^16^. As an alternative, Continuous Glucose Monitoring Devices (CGMs) can infer the blood glucose levels in real-time based on the glucose in the interstitial fluid. These devices significantly empowered diabetic patients, but still, they present some limitations that make them unattractive for pre-diabetic patients and diabetics. Commercially available CGMs can be worn for a limited number of days, usually between 7 and 14 days. Most of the CGMs require finger prick calibration; some studies reported that the reliability of CGMs is limited^17–19^ during low blood glucose level events and they sample from the interstitial fluid which still requires a cannula to be inserted in the subcutaneous tissue, which makes them still invasive.

Moreover, CGMs are quite expensive, which may further limit their use for continuous daily glucose monitoring, especially in pre-diabetic patients. However, despite these potential caveats, recent research revealed that CGM systems overcome the limitations of the self-monitoring of blood glucose (SMBG) using glucometers by providing a complete glucose profile and a detailed history of the nocturnal glucose levels. Therefore CGMs are improving glucose control in diabetic patients^19–22^. International standards for reliability of the SMBGs have been designed (i.e., ISO 15197:2013), while similar standards for CGMs have not been published yet. A number of metrics have been proposed to characterise the accuracy of the CGMs and one, in particular, has emerged as being the most recurrent measure for the sensor accuracy, which is the mean absolute relative difference (MARD). Different studies reported MARD values of 9.5% to 19% for different CGM sensors^17,19–26^, which are close to the values reported for glucometers (5.6% and 20.8%)^27^.

Several non-invasive (e.g., without skin penetration) technologies have been proposed; usually, they are referred to as non-invasive continuous glucose monitors. These devices leverage techniques such as raman spectroscopy^28^, fluorescence technology^29^, optical coherence tomography^30^, optical polarimetry^31^, all aiming to exploit the changes in the chemical and physical tissues properties determined by the glucose variations. Recent reviews^31–33^ of these devices showed that they are promising, although the underlying technology might be improved, making them more accurate, more comfortable to wear, operate, maintain and calibrate.

Finally, the majority of CGMs technologies are not yet inner designed to combine glucose measurements with other physiological signals or activity measures, which may reflect the subject physical and emotional conditions.

The increased number of wearable non-invasive sensors developed for tracking activity or cardiac signal (e.g., ECG) are creating new and unexplored opportunities for early detection of hypoglycemic events. New strategies have been proposed to overcome the limitations of the CGM devices such as: combining direct glucose data with physiological parameters to improve the accuracy of the readings (i.e., enhanced-direct CGM)^34–38^. Other studies combined physiological parameters, vital signs, food intake for the estimation or prediction of either glucose levels or hypoglycemia/hyperglycemia events (i.e. indirect CGMs)^39,40^.

The use of ECG data to detect or predict hypoglycemia (i.e., minimally-invasive indirect CGM)^41–44^ has also been proposed; however, only using non-personalised approaches. The ECG-based systems for hypoglycemia detection are very promising as ECG can be recorded, transmitted and processed quite easily and ECG sensors can be embodied in every-day-use objects (e.g., car steering, the backrest of an office chair, or smartwatches). Moreover, ECG-based glucose detection can be more cost-effective and attractive for pre-diabetic individuals or patients suffering from other comorbidities, who may be familiar with ECG monitoring applications both for clinical and consumer (e.g., sport, fitness) applications.

Associations between ECG parameters (e.g., mainly the QT interval duration) and glucose levels have been investigated in both healthy and diabetic subjects^45–50^ in the past years. It is known that blood glucose concentration can affect the electrical activity of the heart, although the mechanisms behind these changes are not yet completely understood, according to Lipponen *et al*.^48^. Two reported mechanisms are hypokalemia and the disruption of the neural regulation system. Hypokalemia increases potassium conductivity in the myocardial tissue resulting in shorten action potentials. This is known to affect the ECG, causing ST depression, biphasic T-wave (first positive, then negative) followed by a positive U-wave^51^. Both hypokalemia and neural regulation are fast, and thus changes in the ECG should be coincident with the occurrence of the low blood glucose levels^48^. A third possible cause is that low blood glucose levels affect the hormonal secretion, which will determine a delay in the cardiac changes with respect to the onset of hypoglycemia^48^.

A variety of methods have been proposed to detect low-glucose levels using different combinations of ECG features, including principal component analysis (PCA)^52^, genetic algorithms^44^, particle swarm optimization^53^, neural networks^54^. However, none of those studies proposed a model, tool or method to detect low-glucose levels using the raw ECG waveform automatically. Commonly, the used ECG features include QT interval, RT-amplitude ratio and heart rate (HR)^43^. HR, QT interval, change of HR and change of QT were used as inputs in a system for hypoglycemia detection in type 1 diabetic children based on extreme learning machine (ELM) methodology^41^.

ECG feature extraction suffers from high sensitivity to ECG anomalies such as significant changes in the T-wave morphology (e.g., flat or inverted) as a reliable measurement of QT is not straightforward^43,52,55^. Moreover, the majority of the studies investigating ECG-blood glucose associations have been carried out in a controlled clinical setting and not in real-life conditions. Several studies which investigated the hypoglycemia effects on ECG recruiting healthy participants induced low-glucose levels using the clamping technique^56^, in order to bring the blood glucose concentration to values between 3 mmol/L and 3.5 mmol/L^43,45,48,52^. Also, those studies reported as main limitations the difficulty of handling ECG anomalies (mainly changes in the T-wave morphology) and the small number of participants, especially in relation to high differences in individual ECGs^57,58^. Besides, all these approaches required heavy crafting on data preprocessing and feature extraction, selection and prioritisation.

Recently, significant effort has been spent on exploiting deep neural networks, mainly convolutional neural networks (CNNs) and recurrent neural networks (RNNs) for time series classification as a general task, including multi-channel CNNs for multivariate time series classification^59^, multi-scale CNN for univariate time series classification^60^, a fully CNN for time series classification^61^. Those applications consistently demonstrated improved results over traditional machine-learning methods that rely on predefined, manually-extracted features^62^. Consequently, DL applications for physiological time series are growing exponentially. Among the most successful applications that exploit the ECG signal with DL are the ECG classification, arrhythmia detection, screening for proximal atrial fibrillation or cardiac contractile dysfunction^63–68^.

This paper presents the results of a pilot project aiming to develop a personalised DL system for automatic nocturnal low glucose level detection in healthy individuals, based only on ECG acquired with wearable devices in every-day living conditions. This study followed a personalised medicine approach in response to the existing literature that shown that hypoglycemia detection systems trained on cohorts of participants failed to capture the inter-subject variability in the ECG and the ECG changes induced by hypoglycemia. Finally, the proposed method helps clinicians visualising the peculiar ECG changes that are the most informative for automatic detection of low glucose levels in each individual, making the proposed method intelligible

## Results

### Classification of ECG signal that corresponds to normal/low glucose values

We aimed to detect the low glucose levels in healthy individuals based on the ECG signals and actigraphy, recorded continuously during a nominal period of 14 nights for each subject. ECG, actigraphy and CGM were recorded using commercial wearable sensors. A total number of 8 healthy participants were recruited, of which 4 met the inclusion criteria (i.e., at least 2 hypoglycemic events lasting more than 5 minutes in at least 2 nights). In fact, 4 participants did not experience any hypoglycemic events during the recording period, which was not surprising as participants were healthy subjects. A CNN network was trained on the isolated heartbeats extracted from the raw ECG signal, as detailed in the Methods section. The proposed system is a person-specific one in which data recorded for a participant during the first days, were used for training the model, which was tested using data from the same subject acquired in the remaining days. However, there were a few exceptions when the occurrence of low glucose events was not balanced during the recording period, so the days considered for training and testing were not consecutive. In this study, we assumed that the cardiac changes occurred in the same time as low blood glucose levels, and we did not account for other lag except for the 5-minute delay in glucose levels readings introduced by the CGM, as reported by the manufacturer. Actigraphy was measured using commercial sensors embodied in the body-worn ECG device. Activity levels were estimated from the 3-axis accelerations and computed as $VMU=\sqrt{{x}^{2}+{y}^{2}+{z}^{2}}$ where x, y and z are the averages of the three-axial acceleration over the previous 1 second. Thus, the detection algorithm was mathematically formulated as following:

$$GlucoseLevel=f(ECG\_beat,\,Activity\_level)$$

**Figure 1:**
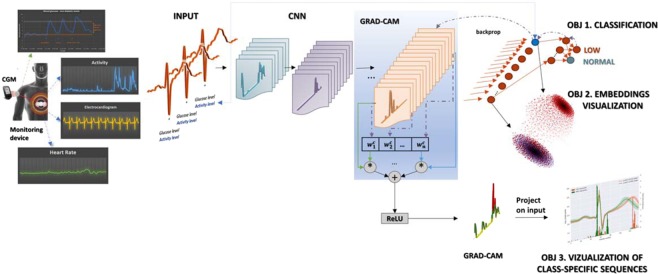
Proposed CNN based system illustrating the study objectives. To detect the low glucose levels using the ECG signal, we set three objectives: (OBJ 1) was to build a classifier (using a CNN network) for the low glucose levels detection task. Secondly, the chosen method for performing the classification (i.e., CNN) enables to investigate further the learned representation of the input heartbeats (OBJ 2), representation (embedding) that can be used in for data visualisation/clustering in lower-dimensional space. The method used for the nonlinear dimension reduction is t-SNE^71^. The third objective (OBJ 3) was to investigate the important regions in the input time series (the heartbeat signal) that contribute the most to the final classification result (Grad-CAM method).

**Figure 2:**
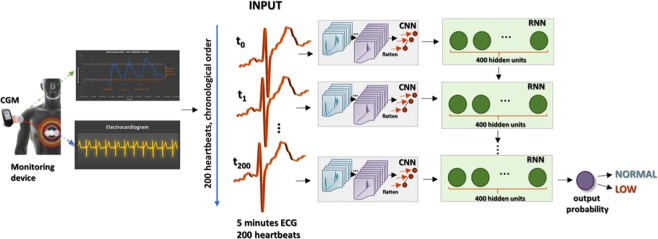
Proposed CNN + RNN system for low blood glucose detection in a 5-minute window of time. The individual heartbeats were firstly isolated, then grouped into 5-minute segments. Each considered 5-minutes segment was chosen if it contained at least 200 heartbeats. This condition also implies that the glucose event (low/normal) should last for at least 5 minutes, thus each 5-minute ECG segment was associated with a single label: low/normal glucose. Each heartbeat was firstly transformed into a feature representation using a CNN network, representation that was fed as input to the sequence model (RNN cells). The outputs of the final RNN are the inputs to a linear layer with a softmax producing a distribution P over the two possible outputs: normal or low glucose values.

The low glucose detection problem was cast to a classification problem in which the inputs represent the extracted heartbeats together with an additional covariate - the activity level and output – normal/low glucose level. To test the feasibility of heartbeat classification by glucose levels, we propose two different approaches: a CNN based system as presented in Fig. 1 and a CNN + RNN system, shown in Fig. 2, models described in more details the Methods section. The reason behind the CNN choice lies in their capability of learning hierarchical, abstract representations of the input space that are relevant to performing specific tasks. The CNN + RNN model was built considering RNN’s ability of learning sequences, in our case sequences of consecutive heartbeats, which were supposed important for detecting low glucose events. In this combined model, the CNN module was used for learning the heartbeat representation, while the RNN component was responsible for learning the heartbeats sequence in the considered 5 minutes ECG intervals. Following personalised medicine approach, we trained the two models (CNN, CNN + RNN) from scratch for each participant using a variable number of recording nights out of which at least 2 nights should contain low blood glucose events. The number of final heartbeats included in the training/validation and testing was reported in the Methods section.

### CNN based system

**Figure 3:**
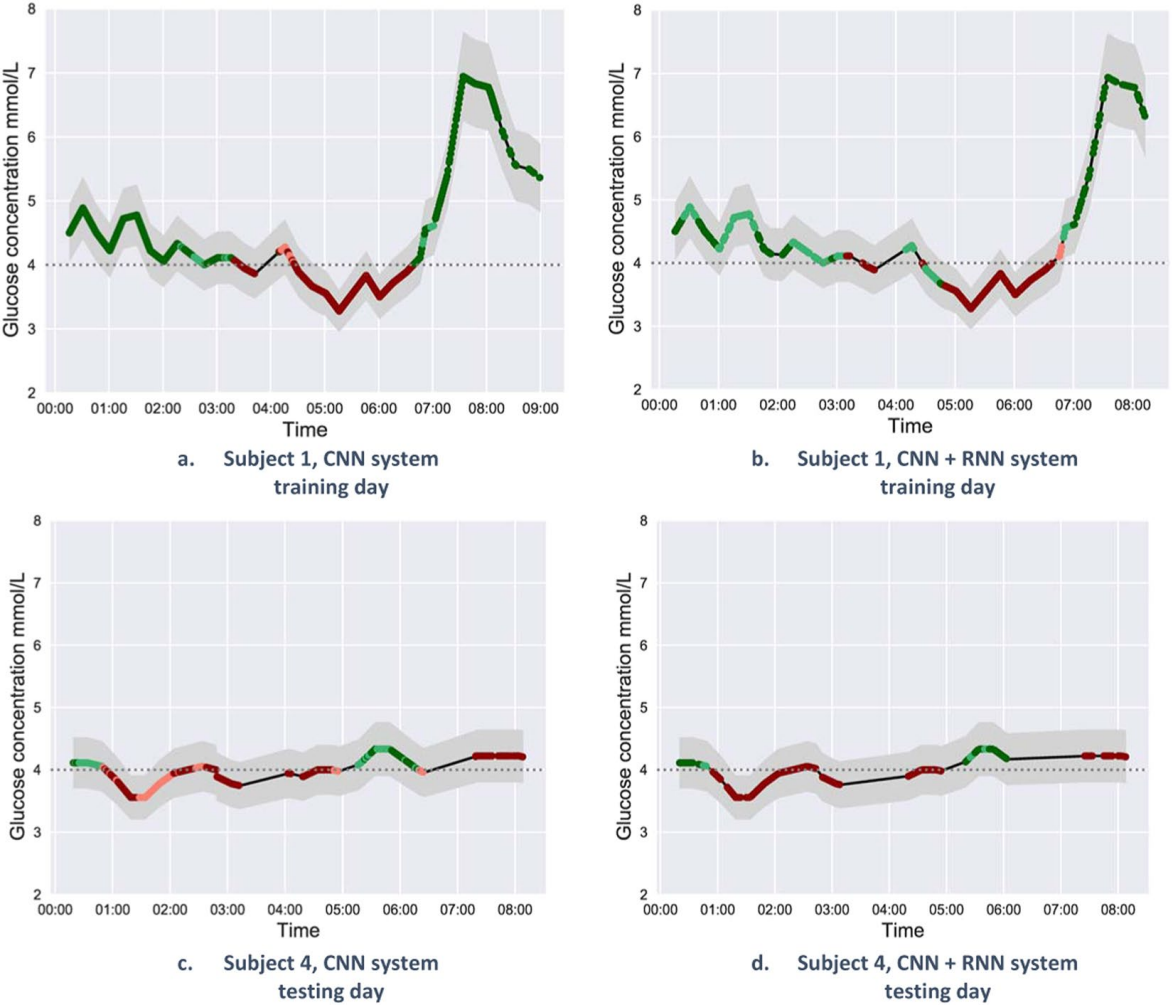
Hypoglycemia detection during the night using the heartbeat majority voting in the 10-minute window of time. The black waveform represents the glucose values recorded by the CGM, considered as ground truth glucose level in this study. The grey shaded regions illustrate a ± 10% error boundary for the CGM glucose readings^18,19^ as it has been reported in previous studies. The colour of the points indicates the predicted class: red for the predicted low-glucose levels and green for the predicted normal-glucose levels. Moreover, dark colours indicate more certain predictions: dark red points accounted for low-glucose predictions with the predicted probability > 0.7, while light red accounted for low-glucose prediction with predicted probability ≤ 0.7; dark green accounted for normal-glucose prediction with predicted probability > 0.7 and light green accounted for normal-glucose prediction with a probability ≤ 0.7. Images a and b present the glucose levels predictions for a sample training day, while columns c and d present the glucose predictions for the same sample test day. The missing heartbeats are due to one of the following reasons: participant removing the sensor, artifact due to movement; missing glucose data, ECG pre-processing.

For the CNN based system, we considered two evaluation strategies, one in which we classified all the individual heartbeats corresponding to the test days and a second strategy used was to employ a majority-voting scheme for the heartbeats in a 10-minute window of time. Results are reported in Table 1 for all the eligible participants on the testing and training datasets. Figure 3 presents the same results for training (a) and testing (c), but visualised as predicted events over the night, in direct comparison to the baseline CGM glucose values. This visualisation provides insightful information about the predicted glucose events otherwise disregarded, including what time certain events occurred, what time the misclassified events occurred, the connection between misclassified events and the considered low glucose threshold, the lag between CGM glucose readings and predictions. Moreover, Fig. 3 reveals the certainty of the model’s predictions, indicated by different colour intensities (dark green/red for most certain prediction), CGM readings uncertainty (grey shadow around the continuous line) and proximity to the glucose level threshold. As expected, the majority of misclassification events and the less-certain classifications (light green/orange) occurred when the glucose levels were close to the threshold with the grey shadow crossing it. The results presented in Table 1 reveal that even when the number of normal glucose heartbeats greatly exceeded the number of low glucose heartbeats (10 times more), the proposed system was still providing a good result, suggesting that DL was resilient to the unbalanced dataset. This was the case for participants 2 and 3 (Table 1).

**Table 1:** CNN based model classification results, evaluated for each participant(testing dataset and training dataset).

Subject id	Sensitivity %		Specificity %		Accuracy %		Number of correctly predicted 10 minutes intervals/total
	Individual beat	10 min voting	Individual beat	10 min voting	Individual beat	10 min voting	
**NN based system classification results, evaluated on testing data for each participant**							
Subject 1	74.2	78.0	71.2	77.1	72.3	77.4	106/141 = 75.2%
Subject 2	66.0	79.8	69.5	77.1	69.3	77.3	146/187 = 78.1%
Subject 3	82.2	100	87.4	91.9	87.1	92.4	168/183 = 91.8%
Subject 4	81.1	91.5	76.3	80.5	77.2	82.6	128/156 = 82.1%
Average	75.9 ± 7.4	87.5 ± 10.3	76.1 ± 8.0	81.7 ± 7.0	76.5 ± 7.7	82.4 ± 7.0	81.8%
**CNN based system classification results, evaluated on training data for each participant**.							
Subject 1	91.3	94.6	79.7	80.4	84.8	86.5	118/136 = 86.8%
Subject 2	93.1	100	87.5	93.2	88.5	94.5	174/184 = 94.6%
Subject 3	97.5	100	88.4	89.7	89.9	91.5	247/270 = 91.5%
Subject 4	75.0	83.5	81.3	85.5	78.3	84.6	138/163 = 84.7%
Average	89.2 ± 9.8	94.5 ± 7.7	84.2 ± 4.3	87.2 ± 5.5	85.4 ± 5.1	89.3 ± 4.5	89.4%

The individual beat column presents the results for each heartbeat classification, whereas the 10-minute voting column shows the results when taking the majority class corresponding to all heartbeats in a 10-minute window of time.

### CNN + RNN based system

The CNN + RNN system was evaluated on 5-minutes input ECG excerpts. The first extracted 200 heartbeats in the 5 minutes ECG were considered as input sequences for the CNN, the output representation of the CNN was fed into a stack of RNN cells, which produced the final prediction, as shown in Fig. 2. To evaluate the model, a majority voting was performed for 10 minutes ECG segments, similar to the voting employed for the CNN-based model. The voting strategy ensured that the prediction frequency was similar to the resolution of the CGMs, which is usually between 5 and 15 minutes, and and it aims to correct the annotation of certain isolated ECG heartbeats. Results are reported in Table 2 for the test and training days, respectively. Figure 3(b,d) presents the classification results, showing the CNN + RNN model predictions over the analysed timeframe for a sample night for two of the subjects.

**Table 2:** CNN + RNN classification results, evaluated on test days for each participant (Testing dataset and training dataset).

Subject id	Sensitivity %		Specificity %		Accuracy %		Number of correctly predicted 10 minutes intervals/total
	5-min ECG	10-min voting	5-min ECG	10 min voting	5-min ECG	10 min voting	
**Classification results evaluated on the testing days**							
Subject 1	79.7	80.5	69.4	73.3	73.3	76	61/82 = 74.4%
Subject 2	81.8	81.8	82.2	88.0	82.2	87.5	154/178 = 86.5%
Subject 3	82.4	76.5	89.6	94.6	89.2	93.7	166/179 = 92.7%
Subject 4	100	100	81.1	82.0	84.8	85.6	128/153 = 83.6%
Average	86.0 ± 9.4	84.7 ± 10.4	80.6 ± 8.3	84.5 ± 9.0	82.4 ± 6.7	85.7 ± 7.3	84.3%
**CNN + RNN classification results evaluated on the training days**.							
Subject 1	74.8	75.2	84.6	88.6	80.0	82.3	78/95 = 82.1%
Subject 2	85.9	84.1	92.8	96.3	91.4	94.1	170/180 = 94.4%
Subject 3	100	100	84.6	89.2	87.2	91.1	240/267 = 89.9%
Subject 4	92.6	90.2	91.9	94.6	92.2	92.6	145/158 = 91.8%
Average	88.3 ± 10.6	87.4 ± 10.4	88.5 ± 4.4	92.2 ± 3.8	87.7 ± 5.5	90.0 ± 5.2	89.6%

The 5-min column presents the results for the 5-minutes ECG segments used during training, whereas the 10-minute voting column shows the results when taking the majority class corresponding to all heartbeats in a 10-minute window of time.

### Localisation of the discriminative subsequences in the input time series, using Grad-CAM

**Figure 4:**
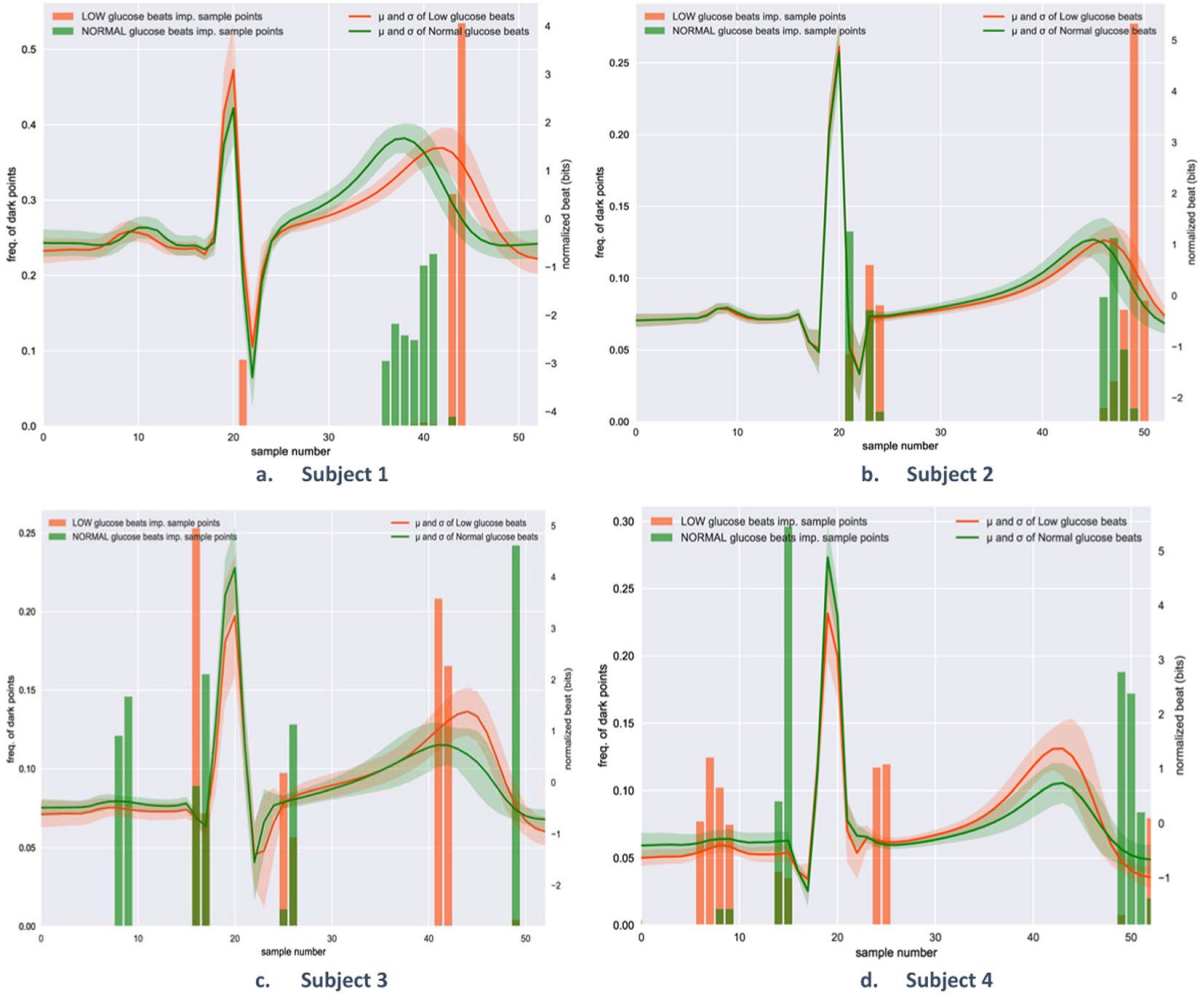
Identification of the most relevant heartbeat segments for hypoglycemia detection using Grad-CAM method. The solid lines represent the mean of all the heartbeats that correspond to each subject in the training dataset: green during normal glucose levels, red during hypoglycemic events. The comparison among 4 different subjects highlighted the fact that each subject may have a different ECG waveform during hypoglycemic events for instance Subjects 1 and 2 present a visibly longer QT interval during hypoglycemic events, differently from subjects 3 and 4. The error bands represent the standard deviation of the considered heartbeats. The vertical bars represent the histograms of the sample points that were >0.9 in the normalised heatmaps obtained from applying Grad-CAM methods on all the training heartbeats.

Grad-CAM, as presented in Selvaraju *et al*.^69^ allowed visualisation of class-discriminative sequences in the input heartbeat, without requiring modifications to the CNN architecture or retraining. Figure 4 presents the histograms of the important sample points in the input heartbeats when Grad-CAM was employed. The highlighted subsequences in the input heartbeats were essential information transmitted through the network, enabling the inspection of the class-discriminative information in the input time-series. Therefore, Fig. 4 illustrates the subsequences in the input heartbeats that were the most important for the CNN in the classification. As expected, the onset and the offset of the T wave was mainly highlighted as important in all subjects. P wave was indicated as important especially when the amplitude of the P wave was lower for the low glucose beats than for the normal glucose beats, in subjects 3 and 4. Moreover, the QRS onset and offset were marked as important in subjects 2–4.

### Dimensionality reduction – CNN based system

**Figure 5:**
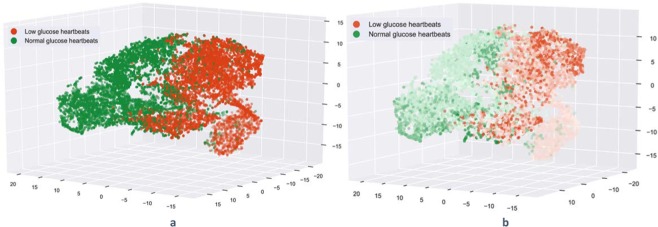
t-SNE visualisation of the test heartbeats corresponding to subject 3 in the activation space representation. The red heartbeats correspond to a low glucose level (<4.0 mmol/L) and the green heartbeats that correspond to normal glucose levels. a. t-SNE visualisation when the heartbeats are coloured according to the glucose annotation(class) b. t-SNE visualisation using a colour map that shows the glucose value associated with each heartbeat, the darker the colour the higher the glucose value for normal beats and the lower for low glucose beats.

The assumption that deeper convolutional layers capture higher-level features, thus that the last convolutional layer should contain the most informative features was extensively explored in previous studies^70^. However, the activation space of CNN’s last layer was too high dimensional for visual investigation and human interpretation. Accordingly, we used a non-linear dimension reduction method to visualise the learned embeddings (i.e., the last CNN layer activations) in lower-dimensional space. Specifically, we employed t-distributed stochastic neighbour embedding(t-SNE)^71^ method for dimensionality reduction. In our study, t-SNE was applied to a balanced subset of the heartbeats included in the test dataset (the normal glucose heartbeats were randomly downsampled without replacement to match the number of the low glucose heartbeats). Figure 5 presents the t-SNE visualisation of the test heartbeats corresponding to one of the subjects (subject 3) applied to the activation space of the CNN’s last layer. In this space, as reported in Fig. 5, it can be observed that the heartbeats are organised in two clear clusters that correspond to the low and normal glucose levels. Moreover, Fig. 5b. shows that heartbeats corresponding to lower (i.e., dark red) or higher (dark green) glucose values are clustered in smaller regions. This could be interpreted as an inner validation of the method proposed and a demonstration of the discrimination power of the network’s learned features, which evidently allowed the unsupervised separation of the heartbeats in two different groups that are also in agreement with the corresponding glucose value magnitude.

### Statistical analysis

The Mann-Whitney rank test revealed that for all the extracted ECG features corresponding to low and normal glucose heartbeats there were significant differences (p-value < 0.01), detailed results presented in the Supplementary material, Fig. 3. The inter-subject statistical tests showed that the ECG features extracted from low and normal glucose heartbeats were also significantly different between subjects, as shown in Figs. 1 and 2 in the Supplementary material. Comprehensive results of this analysis can be found in the Supplementary material, Table 3 and Figs. 1 and 2, and confirmed the hypothesis that a personalised approach was required.

## Discussion

The results of this study have shown that hypoglycemic events can be automatically detected using a few ECG heartbeats recorded with wearable devices in free-living conditions using personalised classifiers based on deep-learning artificial intelligence algorithms. Those results confirmed that heartbeat morphology and the sequence of heartbeats can be used effectively for low glucose detection during the night, in everyday-life conditions. These findings are aligned with clinical studies that presented the predilect cardiac changes occurring during low blood glucose levels, in both healthy and diabetic: prolonged QT interval, increase in the R/T amplitude^45,48,49,72^.

Moreover, comparing the performance of the personalised DL system described in this study with previous cohort-based attempts supported the hypothesis that DL can be used to foster precision medicine. The proposed system can automatically learn patterns in the ECG heartbeat, discriminating between heartbeats recorded during low or normal glucose levels in the same subject. The use of personalised approaches overcame the limits of conventional cohort-based methods accounting for the inter-subject variability in ECG morphology.

As emphasised in the Methods, we restricted our analysis to nighttime recordings, where detection of hypoglycemic events is more useful and also to minimise the influence the circadian rhythm of the cardiac repolarisation that usually lengthens during the night^73^. Our results demonstrate that the proposed CNN based system could accurately detect 10-minutes long low-glucose events with high specificity (average 81.7%) and sensitivity (average 87.5%), as presented in Table 1a. These results advance the idea that no manually extracted features are required to perform this task since deep-learning methods (i.e. CNN) are able to learn automatically highly discriminative features from the raw ECG signals. This is extremely important in the proposed task since feature-based methods are highly dependent upon the correct heartbeat segmentation, where a precise determination of the QT interval would require ECG recording with a high signal-noise ratio that is difficult to measure in real-life and can only be obtained in controlled environments^52^. Thus, the method presented in the current study seems to be particularly useful for real-life settings.

In addition, extra information regarding the sequence of the heartbeats can be captured and presented by combining a CNN with an RNN. The CNN was used to transform the input ECG beats into embeddings that were further aggregated over time by an RNN cell. The predictions for this setup were generated every 5-minutes as the input to the CNN + RNN were the extracted heartbeats in a 5-minute ECG excerpt. The results over 10 min showed that the CNN + RNN model performed on average better than the simple CNN based system, considering (Table 2a) sensitivity (average 84.7%), specificity (average 84.5%) and from the visual inspection of the prediction plots presented in Fig. 3. Filtering out the glucose events shorter than 5 minutes might also contribute to the improved performance over the CNN-based system. Disregarding low glucose events shorter than 5 minutes is in agreement with findings that a low glucose event should last for 10 minutes to be considered a true hypoglycemic event and that very short falls in glucose levels do not reveal the related changes in the ECG^48,74^ signal. Moreover, from the visual inspection of the predictions during the night (Fig. 3) suggested that the regions affected by higher uncertainty (i.e. closer to the threshold of 4 mmol/L) were better classified by the CNN + RNN model, which therefore was considered more resilient.

To the best of our knowledge, this is the first study proposing a personalised system to detect low glucose levels in real-life settings, using the raw ECG signal. Thus, a direct comparison of the obtained results with existing literature is not straightforward. Other studies attempted to detect hypoglycemia through non-invasive monitoring using features extracted from the ECG signal. Studies co-authored by Prof. Hung T. Nguyen^41,42,44,53,54,75,76^ involved nocturnal hypoglycemia detection in 15 type 1 diabetic children using different machine learning techniques (extreme learning^41^, hybrid swarm optimization^53^, neural networks^54^, genetic algorithms^44^, and a few others), using as inputs different ECG parameters computed from 5 or 10 minutes ECG excerpts, and achieving promissing sensitivity and specificity. For example, the more recent studies, proposed models based on a neural logic approach^76^, obtaining 79.07% sensitivity and 53.64% specificity, deep belief network approach^77^, achieving 80% sensitivity and 50% specificity, models based on extreme learning approach, obtaining 78% sensitivity and 60% specificity. As we already emphasised, direct performance comparison with those studies is not viable as the model we proposed is person-specific, which, in our opinion, explains why the proposed method outperforms the results achieved in previous studies. In fact, as demonstrated in Fig. 4, individual ECG response to low-glucose levels varies significantly among different subjects. This affects the classification performance significantly when trying to build a model that can generalise the discriminative features for different individuals. Also, our study concerned the detection of nocturnal non-induced low glucose levels in healthy individuals; several clinical studies showed that cardiac changes could have different intensities in healthy, type 1 and type 2 diabetic persons^48^.

Another advantage of using CNNs based system for the heartbeats classification is the possibility of producing visual explanations for the network’s decisions, thus making the CNN more transparent. We showed that employing a CNN in conjunction with different techniques such as CAM or Grad-CAM could produce a coarse localisation map by using the gradient of the target class with respect to the feature maps of the last convolutional layer, highlighting the important regions or subsequences in the input time series for making a certain prediction. This was important in order to show to our clinical partners, which segment of the ECG excerpt contained the key information utilised by the proposed DL system. Revealing and explaining how the proposed models reached certain conclusions not only makes the models more transparent but can also disclose interesting information about the underlying physiological data.

Previous studies observed the heartbeat changes associated with hypoglicemic events, but mainly during hyperinsulinemic clamps, allowing thresholds for both normal and low glucose levels to be entirely controlled and set to specific values. For instance, Marques *et al*.^78^ considered 3 mmol/l as hypoglycemic level and 5 mmol/l as euglycemic level in type 1 diabetic subjects, Laitinen *et al*.^45^ considered the same limits but in healthy subjects. The common, statistically significant finding in both studies was the lengthening of the QT interval during hypoglycemia. The results presented in Fig. 4 can be interpreted in agreement with this finding, as in all 4 subjects the T-wave was coloured as being important for both classification tasks (i.e. detecting low or normal levels). T-wave flattening was found to be another significant characteristic of hypoglycemia in Laitinen *et al*.^45^ and few other studies^43,48,52^. Our results reveal that the changes in the T wave amplitude are personal. Subjects 3 and 4 presented a mean T-wave amplitude even higher for low glucose heartbeats than for normal glucose heartbeats. We believe that the reasons for this finding are manifold. Firstly, the considered subjects in the current study were healthy and the experiment was carried out in free-living conditions, thus in very sparse occasions, the glucose levels dropped below 3.8 mmol/l. In connection to this, it has been shown that during spontaneous nighttime hypoglycemia in type 1 diabetic patients, the cardiac repolarisation changes are not that intense as during induced hypoglycemia^73^. Also, the CGM device used in our study to record the ground truth glucose levels were shown to have an overall absolute error difference of 11.4% against capillary blood glucose reference^19^; thus some of the heartbeats could have been annotated incorrectly due to the error in CGM readings. Another limitation introduced by the CGM is the glucose reading lag which was reported to be 4.5 ± 4 min, however, in the current study, we accounted for the reading lag 5 minutes independent of the time, activity or food intake. Further research could be carried out to investigate whether drops in potassium levels are coincident with spontaneous glucose falls in healthy, as it is has been shown that low potassium can also determine the flattening of the T-wave^45,47,73^. Interestingly, Fig. 4 reveals that during low glucose levels, the P-wave is more pronounced in some of the subjects (subject 1 and 4) and that P-wave might be important for low glucose detection. This intra- and intra-subject variation in ECG morphology reinforced the idea that there is a clear need for adopting personalised approaches for ECG-based glucose level detections.

The unsupervised clustering of the heartbeats corresponding to low and normal glucose levels using the t-SNE method, presented in Fig. 5, indicates that the CNN network is capable of automatically learning high-dimensional discriminative features. These results demonstrate that the learned feature space can be used to visualise and organise the input data. Data visualisation techniques such as t-SNE can help to inspect the input data and the model, as the similarity of inputs in the original space (thus of the input heartbeats) is also preserved in the obtained low-dimensional space. Figure 5 shows that the learned embedding can separate the heartbeats according to the glucose level. Moreover, the heartbeats corresponding to the glucose extremes: low and high form clear-defined clusters, showing that for heartbeats confidently associated with a certain class the heartbeats are correctly, further separated.

The statistical tests confirmed the need for the development of personalised hypoglycemia detection systems. Moreover, the results provided additional evidence for the less accurate systems developed in the past, that used a pool of ECG features extracted from a cohort of subjects(~15 subjects) to develop different statistical models for hypoglycemia detection. As expected, the results from our statistical analysis showed that the inter-subjects ECG features differences were statistically significant. Therefore, we argue that accurate hypoglycemia alarming systems based on ECG analysis can be developed using personalised ECG-based representation learning methods. Moreover, the personalised approaches proposed in this study showed significant performance improvement in detecting low glucose events over the previous, non-personalised systems.

Given the compelling performance on detecting nocturnal lower glucose levels events in healthy individuals using the ECG signal, we expect that the deep learning based methods similar to the proposed ones in this study to help advance the understanding of electrocardiographic changes induced by the glucose levels variations. Analysis, as demonstrated here, can lead to a better understanding of the underlying processes that determine specific changes in the input heartbeat during low glucose levels. Our results show strong evidence that ECG alterations are feasible to be used for building a real-time alarming system for low glucose events that occur during the night. The obtained results demonstrate superior performance in detecting low glucose levels in comparison to other similar studies, although due to protocol differences, the results cannot be directly compared.

The person-specific framework we propose for detecting low glucose levels in healthy subjects may be utilised in real-life applications as it involved a few preprocessing steps, and it did not require any expert annotations or feature engineering. Our results showed that leveraging deep learning methodologies for the analysis of ECG in order to detect low glucose events can open new possibilities to develop innovative alarming technologies that might help individuals, especially diabetic patients to better control their blood glucose concentrations. Alerting the user in real-time when glucose levels fall below a critical threshold value will facilitate the management of hypoglycemia events and can prevent the development of other severe, life-threatening episodes. Therefore, the proposed system creates the potential for long-term improvements in clinical outcomes, especially in diabetic patients.

Finally, our pilot study must also be seen in the light of its limitations, which also represent important calls for future research avenues. Firstly, additional tests should be performed on a larger population, including diabetic patients, to validate our results further. Secondly, the proposed framework can be easily extended to include other physiological signals that might influence the glucose variation such as activity levels, temperature, skin conductivity or nutrition information that might further improve the performance of the system. For diabetic patients, that will use finger pricks to check their actual blood glucose levels, implementing online training techniques is essential, as the system should be able to also learn continuously, from new data.

The findings presented in this manuscript have been further investigated in a study by the same authors^79^, carried out in a calorimeter room at the University Hospitals Coventry & Warwickshire on eight, healthy, elderly participants. Moreover, the work presented in this research is subject to a patent application Number GB1912487.4, date 30 August 2019. (M. Porumb & L. Pecchia, ‘Electrocardiogram-based blood glucose level monitoring’).

## Methods

### Protocol

Eight healthy volunteers that were not taking any medication, were monitored without any constraints on diet or lifestyle between 8 and 14 consecutive days. More information about the participants’ demographics and their glucose profiles can be found in the Supplementary material, Tables 1 and 2. Four participants were excluded from the study, due to a shortage or lack of hypoglycemic events, as defined in the exclusion criteria presented in the following paragraphs. The study protocol was approved by the Ethics Committee of the University of Warwick, UK (REGO-2018-2205), and each person enrolled gave written informed consent to participate. The authors confirm that all the experiments were performed in accordance with relevant guidelines and regulations.

Nominal 24 h ECG was recorded with wearable commercial devices (Medtronic Zephyr BioPatch™ HP^80^). This CE Marked device works with 250 Hz sampling frequency and ECG amplitude between 0.25 and 15 mV. We used this wearable sensor in previous study for similar monitoring (i.e., 24 hours for several consecutive days). Moreover, the Zephyr Biopatch has been used in several clinical trials, assessing its performance as a remote patient monitoring device^81,82^. The ECG monitor can store up to 3 days of ECG recordings, its battery can last for 36 hours and can be fully charged in less than one hour. Therefore, each volunteer was given two devices and instructed to change it approximately each 24-hours before showering. The Zephyr records also 3-axis accelerations and breathing waveform. Based on the raw accelerations an activity parameter was computed and logged by the device @1 Hz, measured in vector magnitude units (VMU), a parameter that was also included in the proposed framework in addition to the ECG signal.

Continuous glucose levels were measured using FreeStyle Libre Flash glucose monitoring system^83^, which measures the interstitial glucose every 15 minutes. Each glucose sensor can be used for up to 2-weeks, also while showering, and according to the producer does not require any calibration with finger pricks. The recent development in CGM technology^19,84^, enabled us to use the factory-calibrated flash glucose monitoring system (FreeStyle Libre) as baseline glucose levels readings in this study, given the real-life requirement. The FreeStyle Libre system is clinically proven to be accurate, stable and consistent over 14 days compared to blood glucose testing without the need for finger-prick calibration^19,26,85,86^. In a clinical study involving 72 type 1 and type 2 diabetic patients, the FreeStyle Libre system achieved 11.4% Mean Absolute Relative Difference (MARD) compared to blood glucose testing and 99.7% of glucose results fall within Zone A and Zone B of the Consensus Error Grid, when compared against blood glucose testing^19^. A comparative study assessing 17 point-of-care glucose meters, showed that the accuracy varied widely from 5.6% to 20.8% MARD, therefore providing evidence that the CGM accuracy is comparative to the accuracy of the point-of-care glucose meters. Moreover, in July 2018 the Food and Drug Administration (FDA) approved the FreeStyle Libre device, the decision came after Abbott published a clinical trial involving 95 subjects, which found that patients who used the scanner frequently had improved glycemic control and less hypoglycemia, reporting an overall MARD of 10.1% compared to blood glucose testing^21^. Moreover, the CGM readings in this study were used to assess whether the glucose levels dropped below a threshold. Therefore, the CGM readings were used to make a dichotomic decision and not for determining the precise glucose values, which enabled us to use a CGM readings as baseline for the ECG classification. To further account for the potential CGM readings inaccuracies, the heartbeats corresponding to a glucose level > hypoglycemia threshold and <hypoglycemia threshold + 0.5% were not considered during training.

During the 24 hours, the ECG sensor was typically removed during showering and during high-intensity activity (usually training/workout) which may cause the electrodes to loosen due to sweat or movement of the sensor which can also introduce extra noise in the ECG recordings. Therefore, the available ECG data is variable for each participant during a 24 hours window of time. Some glucose readings might also be missing as the sensor requires to be scanned at least once every 8 hours, in case of a missing scan, the data that exceeded 8 h was not logged. Moreover, the first and the last days of recordings were disregarded from the analysis as studies that investigated the CGM performance showed that the accuracy of the glucose recordings is the lowest in the first day and that it also decreases towards the end of the recording period^19^.

Furthermore, as mentioned earlier, this study concerns the detection of nocturnal (midnight to 9 AM) low glucose events, although the continuous ECG and glucose recordings were collected almost continuously during a 24 h period. There are two main arguments for this choice. Firstly, only in few participants (two), the recorded glucose levels dropped below the considered low threshold during the daytime, but still the available low events were not enough in order to develop and validate the proposed system. Secondly, it is known that the cardiac repolarisation has a circadian cycle that usually lengthens during the night^73,87–89^, therefore it is essential to consider whether the ECG changes reflect some circadian physiological alterations or they are indeed induced by the lower blood glucose concentrations. Therefore, due to the expected ECG circadian changes and the shortage of low glucose events during the day, we decided to consider for the analysis only the data that was recorded during the night. To ensure that the low glucose detection model does not capture just the associated circadian ECG changes, we limited the analysis in this study to the night period.

### Dataset

ECG excerpts of 15 minutes were annotated as corresponding to normal or low glucose according to the CGM readings. Since our study focused on healthy participants monitored in real life (i.e., no induced low levels via clamping), lower glucose level (i.e., LGL) episodes were defined as glucose concentration values lower than 4 mmol/L. A normal glucose level (NGL) was defined as a glucose concentration between 4 mmol/L and 7.5 mmol/L, as per international guidelines^90^. For a subject to be included in the analysis, at least 10% of their recorded glucose values were expected to be less than the LGL threshold plus a small error of ~0.2 mmol/L (to account for the CGM reading error), thus less than 4.2 mmol/L. Moreover, the glucose value that corresponds to the 80^th^ percentile of the recorded glucose values was expected to be less than the NGL threshold. Thus, the percentiles condition represents an additional check that the person is healthy, and that the majority of the glucose levels recorded during consecutive nights lie between the expected values.

The complete dataset comprises of ECG and glucose recordings for 8 participants that worn the two sensors between 8 and 14 days. Four participants were excluded from the analysis because their glucose levels did not go below the established threshold of 4 mmol/L for the low glucose (subjects 5, 6, 7), essentially, they did not experience low glucose events or very few that were not enough to satisfy the condition that at least 10% of the recorded values should be <4.2 mmol/L (subject 8). Moreover, after being enrolled in this study subjects 5 and 6 were diagnosed as being pre-diabetic, finding that is also reflected by the high glucose values recorded by the CGM. Therefore, the remaining 4 subjects included in the analysis were subject with IDs 1, 2, 3 and 4.

### CNN based system dataset

The final dataset used for building and testing the CNN based system comprised of a list of ECG heartbeats each having associated 2 additional parameters: an activity level and the corresponding glucose value used as output. To account for the reported average lag time of the FreeStyle Libre system readings, which is known to be approximately 5 minutes^19^, each heartbeat was associated the glucose value that corresponded to the current timestamp of the heartbeat plus 5 minutes. Moreover, the heartbeats that corresponded to glucose levels between 4 and 4.2 mmol/L were not considered during training. This measure ensured that no consecutive heartbeats would be considered as both low and normal, therefore reducing overfitting and accounting for the CGM error close to the chosen threshold for hypoglycemia. For each participant, the recording nights were split into 2 separate datasets for training and testing the model, ensuring that every dataset contained nights with low blood glucose events. An additional validation dataset was created by randomly resampling without replacement 20% of the heartbeats included in the training dataset. The final number of extracted heartbeats for each participant corresponding to the normal and low blood glucose levels is presented in Table 3. When the number of low glucose beats was less than 25% of the number of normal ones during training, the majority class was randomly downsampled without replacement. No other specific methodology (such as oversampling, cost-sensitive learning) was employed for balancing the dataset. The validation dataset was used to monitor the training and to early stop, in case the Area under the ROC curve (AUC) evaluated at every 100 steps did not improve in the next 10 evaluations. The best model as assessed on the validation set was saved during the optimisation process.

**Table 3:** Number of extracted heartbeats from the nighttime ECG recordings for each eligible participant enrolled in the study and their distribution in training and testing datasets.

Subject ID	Number of heartbeats				Number of recording nights (nights with low glucose events)	
	Training		Testing		Training	Testing
	Normal	Low	Normal	Low		
1	37042	38798	35991	22216	3 (3)	3 (3)
2	51026	18266	68941	6321	4 (3)	4 (3)
3	92261	21844	69533	5053	6 (2)	4 (1)
4	28342	34491	46345	13544	4 (3)	4 (2)
Average	52168	28350	55203	11784	4.3 (2.8)	3.8 (2.3)

### RNN based system dataset

The same recording nights were used for building and testing the CNN + RNN system. Instead of considering the individual heartbeats as inputs, the inputs into the RNN network represent the sequence of the first 200 consecutive heartbeats from a 5-minute non-overlapping ECG excerpt. To ensure that each 5-minute ECG excerpt corresponded to a glucose event: either low or normal, the glucose events that did not last for 5 minutes were filtered out. A low glucose event shorter than 10 minutes, was most probably caused by an inaccurate glucose reading (thus most probably an outlier). Moreover, to ensure that the HR in the 5-minute ECG excerpts > 40 bpm, only those 5-minutes ECG intervals that contained at least 200 heartbeats were included in the analysis. Similarly, the heartbeats corresponding to glucose values between 4 and 4.2 mmol/L were not considered for training the model (CGM reading + 5% error). Table 4 presents the final number of 5-minutes ECG segments that were selected and included in the training and testing datasets.

**Table 4:** Total number of extracted 5-minute ECG excerpts from the nighttime ECG recordings for each eligible participant enrolled in the study and their distribution in the training and testing datasets.

Subject ID	Number of 5-minute ECG excerpts			
	Training		Testing	
	Normal	Low	Normal	Low
1	130	119	118	69
2	275	64	315	22
3	409	86	317	17
4	149	122	217	53

### Data pre-processing

Since this study investigated the association between ECG beat morphology and glucose levels, the first step was to isolate each heartbeat. This was achieved by detecting a fiducial point (i.e., the R peak) and then selecting a window of time of 640 ms around the fiducial point, in analogy to^64^ and accounting for the sampling frequency. The fiducial point for each heartbeat was detected using a QRS detection algorithm as proposed in^91^. Since the ECG were sampled at 250 Hz, a window of time of 640 ms was isolated counting 160 ECG samples around the R fiducial point (i.e., 60 samples preceding the R peak and 100 samples following the R peak). Two parameters logged by the Zephyr BioPatch were used to filter the noisy ECG segments: heart rate confidence and the ECG noise. According to the device’s specification, The HR confidence takes into account a worn detection indication and the signal-to-noise ratio of the ECG signal. In the current study, ECG excerpts with 100% HR confidence and ECG noise < 0.001 were extracted and considered for the beat extraction and further for the analysis. After the heartbeats were isolated, they were z-normalized and downsampled, keeping only the kth sample (with k = 3). Thus, the final length of the heartbeat time series was 53 sample points.

### CNN network

The proposed CNN network was implemented in TensorFlow^92^, it comprises of 15 convolutional layers with a fixed number of 50 filters in each layer, in agreement with previously published models^64^ and one fully connected (FC) layer of 30 neurons. The activity level information that was associated with each heartbeat was also included in the CNN network as an additional neuron in the FC layer, as presented in Fig. 1. The network was trained from scratch, initialising the weights of the convolutional layer as in^93^ using the Xavier initialiser. The sizes of the filters used were kept constant being set to 3, that represents around 5% of the input time series length (53 samples). The employed loss function was the cross-entropy between the estimated class probabilities and the target classes. The chosen optimizer was AdamOptimizer^94^ with an initial learning rate of 1e-4. Batch normalisation was employed after each convolution and before the ReLU^95^ activation. No pooling operation was used except a 0.5 rate dropout after the fully connected layer. The maximum number of training iterations was set to 2.5e + 4, which represents at least 45 epochs considering a mini-batch of 200 input beats, for all the participants.

Due to the high flexibility of the CNN structure and the high number of hyper-parameters, we evaluated a combination of architecture and hyper-parameters in an iterative process, using grid-search and manual tuning. Regarding the architecture structure, we searched over the number of convolutional layers (3 to 20), different filter sizes (from 3 to 20) and the number of filters in each convolutional layer (20 to maximum 100). The learning rate was manually tuned in order to achieve a faster convergence; the considered values were {10^−1^ to 10^−5^}. The results presented in this manuscript were obtained on the final CNN architecture that achieved the highest performance on the validation dataset, that also minimised the number of parameters.

### CNN + RNN network

The proposed CNN + RNN based system leverages the representation power of the CNNs and connects the obtained representations to a recurrent neural network in order to also capture temporal dependencies between the input heartbeats. Specifically, due to the known exploding or vanishing gradients problems in the RNNs, the recurrent block comprises of long short-term memory (LSTM) cells. The CNN + RNN model works by passing each input (individual heartbeat, *b*_*i*_) through a feature transformation *ϕ*_*V*_ with parameters V, which in our case is a CNN network, to obtain a fixed-length vector representation. The outputs of *ϕ*_*V*_(*b*_*i*_)) are then passed into a recurrent sequence learning module (i.e. an LSTM network). The recurrent network in a very general form, has parameters W and maps an input *b*_*t*_ and a previous hidden state *h*_t−1_ to an output *z*_*t*_ and an updated hidden state *h*_*t*_. The final system is instantiated with a sequential input (the consecutive heartbeats extracted from a 5-minute ECG excerpts) and has a static output generated only at the last sequence step, which is the glucose event associated with every 5-minute interval (${b}_{1},{b}_{2},\ldots ,{b}_{200})\to y$ (low/normal glucose). To predict a distribution over the outcomes y, at time step t, the outputs *z*_*t*_ of the sequential model are passed through a linear prediction layer, outputs of which are passed through a softmax function to obtain the final class probabilities.

The CNN module comprises 5 convolutional layers each having 50 filters of size 3. The LSTM module comprises a single LSTM layer with 400 units in each LSTM cell and 200-time steps. The weights initialiser for both CNN and LSTM parameters was Xavier initialiser and all biases were initialised to 0. We trained the CNN + RNN network end-to-end through backpropagation and we found that a higher dropout (0.6) was needed to avoid overfitting. The batch size was 30, the initial learning rate was set to 1e-4 and the used optimiser was AdamOptimizer.

### Performance evaluation

The performance measures used for both models (CNN and CNN + RNN) assessment were accuracy, sensitivity, specificity, and AUC. In addition, from the clinical perspective, sensitivity is considered more relevant than specificity as it shows how well the event was identified (in our case the low glucose events), thus when comparing different models, specificity was considered more important.

When training the model, the inputs of the CNN represent the isolated heartbeats, however, the CNN based model we proposed does not account for the sequence of beats in a specific timeframe. In case of a real-time alarming system, predicting a class for every heartbeat will be undesirable and it might be difficult to follow, instead, generating a prediction every 10 minutes is more feasible and closer to the resolution of the CGM devices. For this reason, we also evaluated the model’s performance in a 10-minute window of time, by taking the majority class of the heartbeat predictions in that specific timeframe. The same voting strategy was also applied to the CNN + RNN model.

### Localisation of the contributing ECG beat subsequences with Grad-CAM

In order to obtain the class-discriminative localisation map in a generic CNN architecture, we employed the Grad-CAM method as described in^69^. The technique implies the computation of the gradient of *y*^*c*^ with respect to feature maps A (in our case the feature maps of the last conv layer) that are global-average-pooled to obtain the weights ${\alpha }_{k}^{c}$ similar to the weights ${w}_{k}^{c}$ computed with the CAM method. The weights ${\alpha }_{k}^{c}$

$${\alpha }_{k}^{c}=\frac{1}{m}\sum _{i}\sum _{j}\frac{\partial {y}^{c}}{\partial {A}_{ij}^{k}}$$

capture the importance of feature map k for a target class c. The Grad-CAM heatmap is obtained as a weighted combination of feature maps. It has been shown^69^ that Grad-CAM is a generalisation of CAM and can be used in conjunction with any CNN architecture with fully-connected layers.

### Visualisation of the data in lower-dimensional space

We used a nonlinear dimension reduction method to visualise the data in a lower-dimensional space, in particular, t-distributed stochastic neighbour embedding (t-SNE^71^). We applied t-SNE to the heartbeat embeddings as obtained from the fully connected neurons in the CNN based model.

### Statistical analysis

A series of key ECG parameters were extracted for all the heartbeats included in the train and test datasets. The extracted parameters included: the amplitude of the Q, R, T waves, the QT interval (measured from peak to peak), the RT amplitude (as a ratio of R-wave and T-wave) and the T wave slope (slope of the line that intersects T wave peak and T wave offset point). As T wave could not be accurately detected for all the extracted heartbeats, the heartbeats that could not be fully segmented were excluded from the analysis. Furthermore, we balanced the number of low and normal heartbeats, by randomly downsampling the heartbeats corresponding to the majority class. The total number of heartbeats included in the statistical analysis for each subject were (N = 29732, 14276, 40642, 30998) corresponding to (subject 1, subject 2, subject 3 and subject 4).

Two non-parametric statistical tests (the condition for normality checked using the Shapiro-Wilk test was violated) were performed to assess both intra- and inter-subject ECG features variability. Therefore, Mann-Whitney rank test was conducted to test the changes in the individual ECG features between low and normal glucose levels. To test the changes in the ECG parameters between subjects, a multi-way Kruskal-Wallis H-test was performed for each ECG parameter for low and normal glucose condition separately. A significant four-way interaction between the four subjects indicated that the ECG feature changed significantly for one or more subjects, without specifically indicating between which subjects the ECG features were significantly different. Therefore, to further investigate the pairwise differences between subjects, a post hoc comparison was performed with a two-way Kruskal-Wallis H-test and Dunn’s test. A p-value < 0.05 was accepted as evidence of statistical significance.

### Programming

The deep learning models were developed in Python employing different libraries such as TensorFlow, Numpy, Pandas, and trained on an Intel Core i7 processor with 32GB RAM. To speed up the training, we also used High-Performance Computing facilities i.e. 4 GPU nodes (each having 2 × NVIDIA Tesla K80 GPU cards) provided by the Centre for Scientific Computing (CSC) at University of Warwick.

## Supplementary information

Supplementary Material.

## Data Availability

The data sets used in this study and the code are available from the corresponding authors on request.
