# Supplementary material for: Precision Medicine and Artificial Intelligence: A Pilot Study on Deep Learning for Hypoglycemic Events Detection based on ECG
Source: Sci Rep. 2020 Jan 13;10:170. doi: 10.1038/s41598-019-56927-5 (PMC6957484; doi:10.1038/s41598-019-56927-5)
Supplement: Supplementary file 1 — Supplementary Material. [file 41598_2019_56927_MOESM1_ESM.pdf]

# Precision Medicine and Artificial Intelligence: A Pilot Study on Deep Learning for Hypoglycemic Events Detection based on ECG.

Mihaela Porumb, Saverio Stranges, Antonio Pescapè, Leandro Pecchia

December 2019

Table 1: Participants' demographics, the eligible participants are highlighted in green.

| **Participant ID** | **Age** | **Gender** | **Weight(kg)** | **Height(cm)** | **BMI** |
|---|---|---|---|---|---|
| 1 | 35 | M | 80 | 180 | 24.6 |
| 2 | 29 | F | 55 | 162 | 20.9 |
| 3 | 32 | M | 81 | 184 | 23.9 |
| 4 | 27 | F | 63 | 167 | 21.7 |
| 5 (pre-diabetic) | 40 | M | 64 | 170 | 22.1 |
| 6 (pre-diabetic) | 56 | M | 98 | 168 | 34.7 |
| 7 | 58 | F | 75 | 158 | 30 |
| 8 | 26 | M | 78 | 183 | 23.2 |

Table 2: Nighttime (midnight to 9 AM) glucose histograms corresponding to all the available participants. The glucose value of the $10^{th}$ percentile correspoding to participants 5,6,7 and 8 is higher (4.9, 7.1, 5.1 and 4.7 mmol/L) than the expected values of 4.2 mmol/L, thus the 4 participants could not be considered for further analysis.

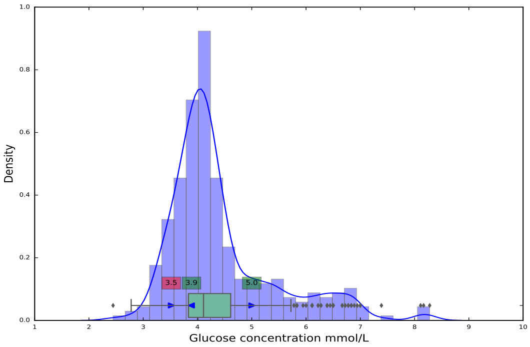


(a) Subject 1, 8 days

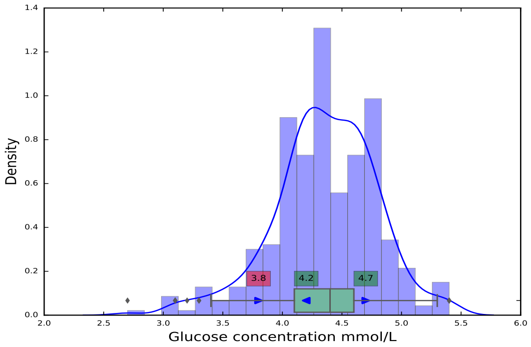


(b) Subject 2, 9 days

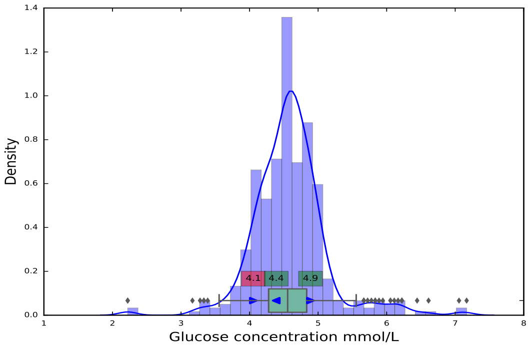


(c) Subject 3, 11 days

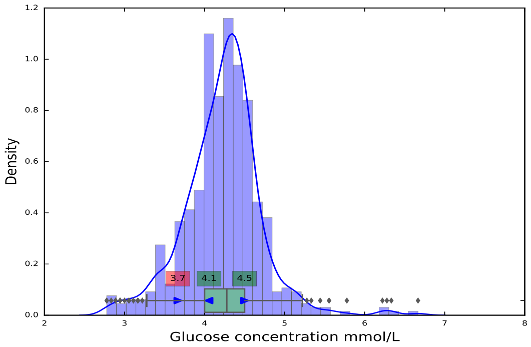


(d) Subject 4, 9 days

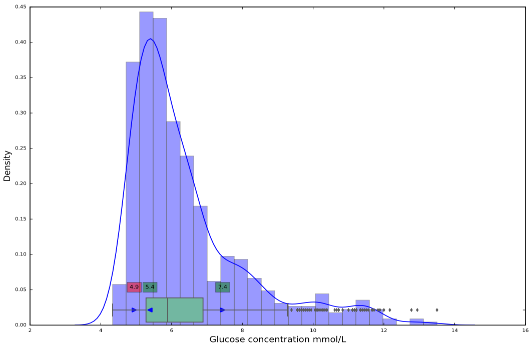


(e) Subject 5, 12 days

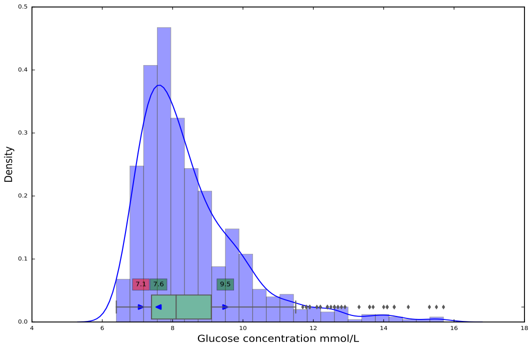


(f) Subject 6, 12 days

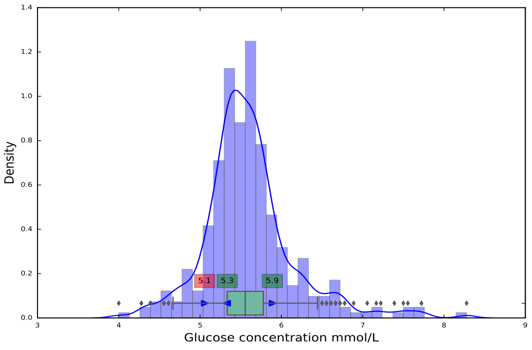


(g) Subject 7, 6.5 days

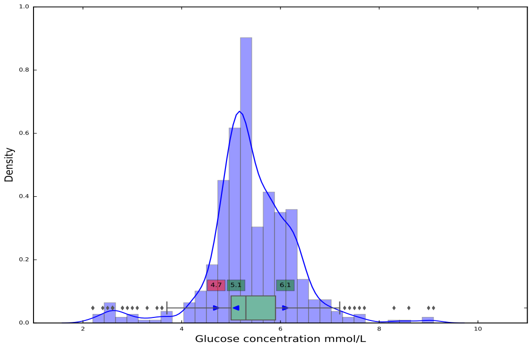


(h) Subject 8, 11 days

Table 3: Kruskal-Wallis H-test between pairs of subjects for the extracted ECG features corresponding to Low glucose (a) and Normal glucose (b). *p-value post hoc* column presents the post hoc pairwise test results for multiple comparisons of mean rank sums using Dunn's test.

(a) Low glucose

| | **Low glucose condition** | | | | | | | | |
|---|---|---|---|---|---|---|---|---|---|
| Group | **Q_amp** | | | **R_amp** | | | **T_amp** | | |
| | H | *p-value* | *p-value post hoc* | H | *p-value* | *p-value post hoc* | H | *p-value* | *p-value post hoc* |
| S1 <->S2 | 8285.5 | **<0.01** | **<0.01** | 13735.6 | **<0.01** | **<0.01** | 9054.44 | **<0.01** | **<0.01** |
| S1 <->S3 | 5390.59 | **<0.01** | **<0.01** | 265.044 | **<0.01** | **<0.01** | 599.2 | **<0.01** | **<0.01** |
| S1 <->S4 | 17489.95 | **<0.01** | **<0.01** | 42.43 | **<0.01** | 0.136 | 293.67 | **<0.01** | **<0.01** |
| S2 <->S3 | 1922 | **<0.01** | **<0.01** | 14554.1 | **<0.01** | **<0.01** | 3659.64 | **<0.01** | **<0.01** |
| S2 <->S4 | 1552.74 | **<0.01** | **<0.01** | 8475.4 | **<0.01** | **<0.01** | 3338.05 | **<0.01** | **<0.01** |
| S3 <->S4 | 10065.08 | **<0.01** | **<0.01** | 372.38 | **<0.01** | **<0.01** | 1075.31 | **<0.01** | **<0.01** |
| | **QT** | | | **RT_amp** | | | **T_slope** | | |
| | H | *p-value* | *p-value post hoc* | H | *p-value* | *p-value post hoc* | H | *p-value* | *p-value post hoc* |
| S1 <->S2 | 13042.9 | **<0.01** | **<0.01** | 11073.3 | **<0.01** | **<0.01** | 365.61 | **<0.01** | **<0.01** |
| S1 <->S3 | 18328.62 | **<0.01** | **<0.01** | 2.98 | 0.084 | 0.064 | 7553.51 | **<0.01** | **<0.01** |
| S1 <->S4 | 1541.51 | **<0.01** | **<0.01** | 53.89 | **<0.01** | 0.064 | 4799.6 | **<0.01** | **<0.01** |
| S2 <->S3 | 4826.29 | **<0.01** | **<0.01** | 5560.2 | **<0.01** | **<0.01** | 3985.8 | **<0.01** | **<0.01** |
| S2 <->S4 | 11073.53 | **<0.01** | **<0.01** | 4097.5 | **<0.01** | **<0.01** | 2501 | **<0.01** | **<0.01** |
| S3 <->S4 | 12925.27 | **<0.01** | **<0.01** | 0.73 | 0.394 | 0.824 | 176.85 | **<0.01** | **<0.01** |

(b) Normal glucose

| | **Normal glucose condition** | | | | | | | | |
|---|---|---|---|---|---|---|---|---|---|
| Group | **Q_amp** | | | **R_amp** | | | **T_amp** | | |
| | H | *p-value* | *p-value post hoc* | H | *p-value* | *p-value post hoc* | H | *p-value* | *p-value post hoc* |
| S1 <->S2 | 9186.49 | **<0.01** | **<0.01** | 11126.85 | **<0.01** | **<0.01** | 4100.15 | **<0.01** | **<0.01** |
| S1 <->S3 | 16000.95 | **<0.01** | **<0.01** | 3072.64 | **<0.01** | **<0.01** | 7111.15 | **<0.01** | **<0.01** |
| S1 <->S4 | 23510.48 | **<0.01** | **<0.01** | 8962.07 | **<0.01** | **<0.01** | 3872.39 | **<0.01** | **<0.01** |
| S2 <->S3 | 3764.68 | **<0.01** | **<0.01** | 11016 | **<0.01** | **<0.01** | 338.98 | **<0.01** | **<0.01** |
| S2 <->S4 | 504.26 | **<0.01** | **<0.01** | 2698.29 | **<0.01** | **<0.01** | 0.07 | 0.798 | 0.948 |
| S3 <->S4 | 17293.89 | **<0.01** | **<0.01** | 2841.9 | **<0.01** | **<0.01** | 22.37 | **<0.01** | **<0.01** |
| | **QT** | | | **RT_amp** | | | **T_slope** | | |
| | H | *p-value* | *p-value post hoc* | H | *p-value* | *p-value post hoc* | H | *p-value* | *p-value post hoc* |
| S1 <->S2 | 10322.81 | **<0.01** | **<0.01** | 6780.41 | **<0.01** | **<0.01** | 1644.39 | **<0.01** | **<0.01** |
| S1 <->S3 | 18477.87 | **<0.01** | **<0.01** | 5800.23 | **<0.01** | **<0.01** | 985.03 | **<0.01** | **<0.01** |
| S1 <->S4 | 23959.46 | **<0.01** | **<0.01** | 7616.6 | **<0.01** | **<0.01** | 1520.74 | **<0.01** | **<0.01** |
| S2 <->S3 | 641.89 | **<0.01** | **<0.01** | 370 | **<0.01** | **<0.01** | 180.86 | **<0.01** | **<0.01** |
| S2 <->S4 | 461.3 | **<0.01** | **<0.01** | 87.68 | **<0.01** | **<0.01** | 0.94 | 0.332 | **<0.01** |
| S3 <->S4 | 58.07 | **<0.01** | **<0.01** | 379.15 | **<0.01** | **<0.01** | 175 | **<0.01** | **<0.01** |

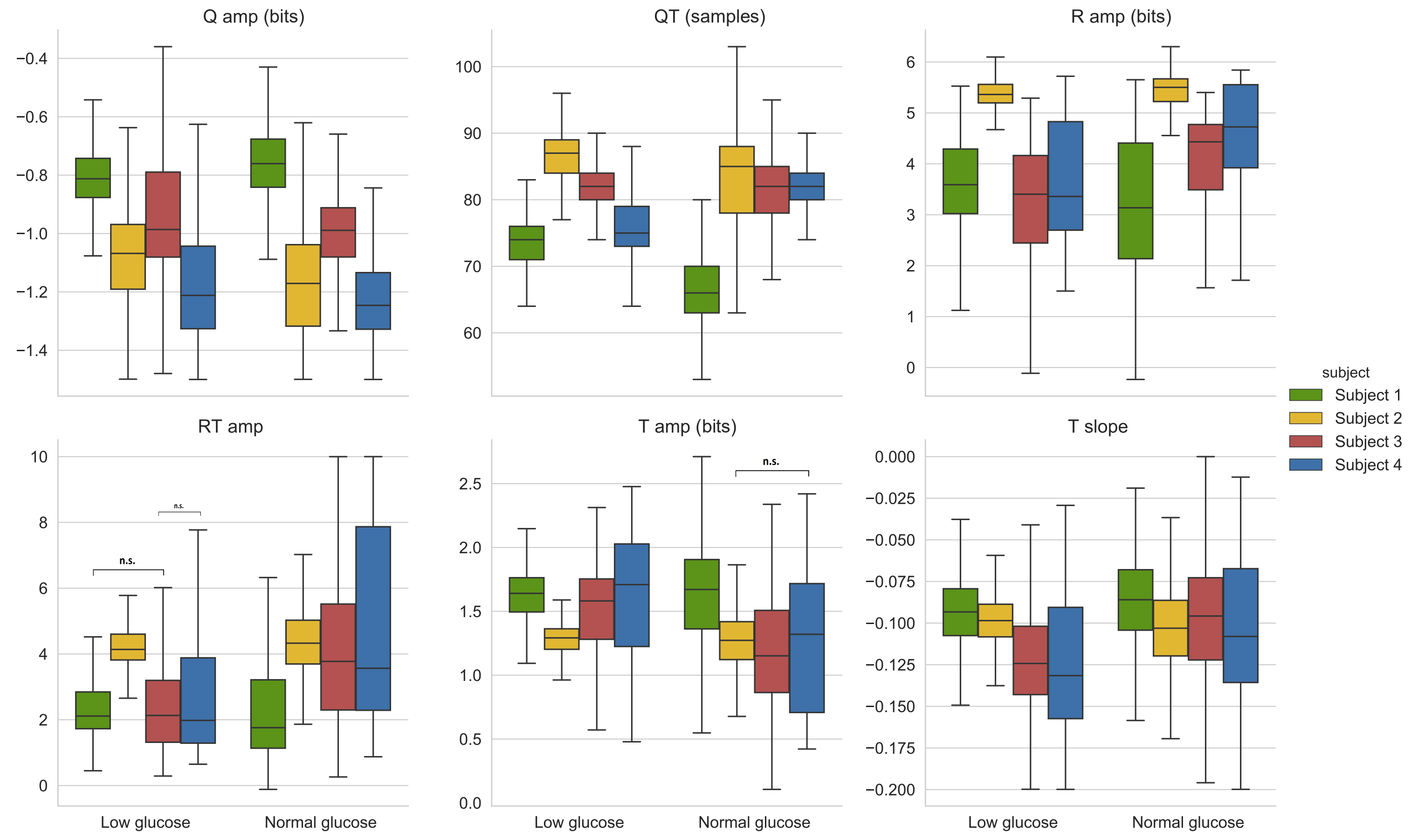


Figure 1: Box plots for the extracted ECG features during Low and Normal glucose levels for every participant. A multi-way Kruskal-Wallis H-test was performed for every ECG parameter for the low and normal glucose condition separately. The only non-significant differences between the groups are indicated in the plot n.s.

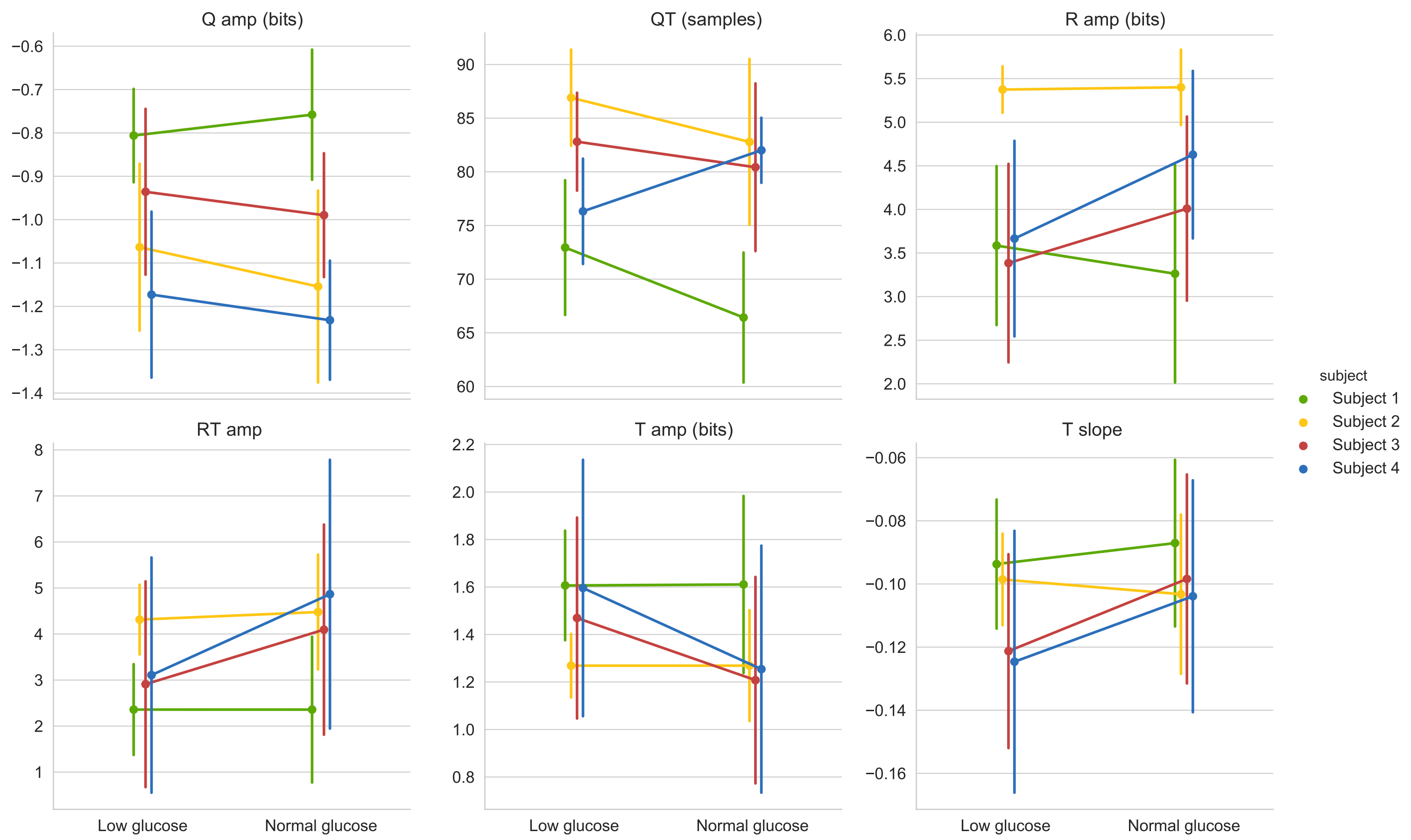


Figure 2: Point plots for the extracted ECG features during Low and Normal glucose levels for every participant, showing the relationship between the mean of every ECG feature for low and normal glucose levels.

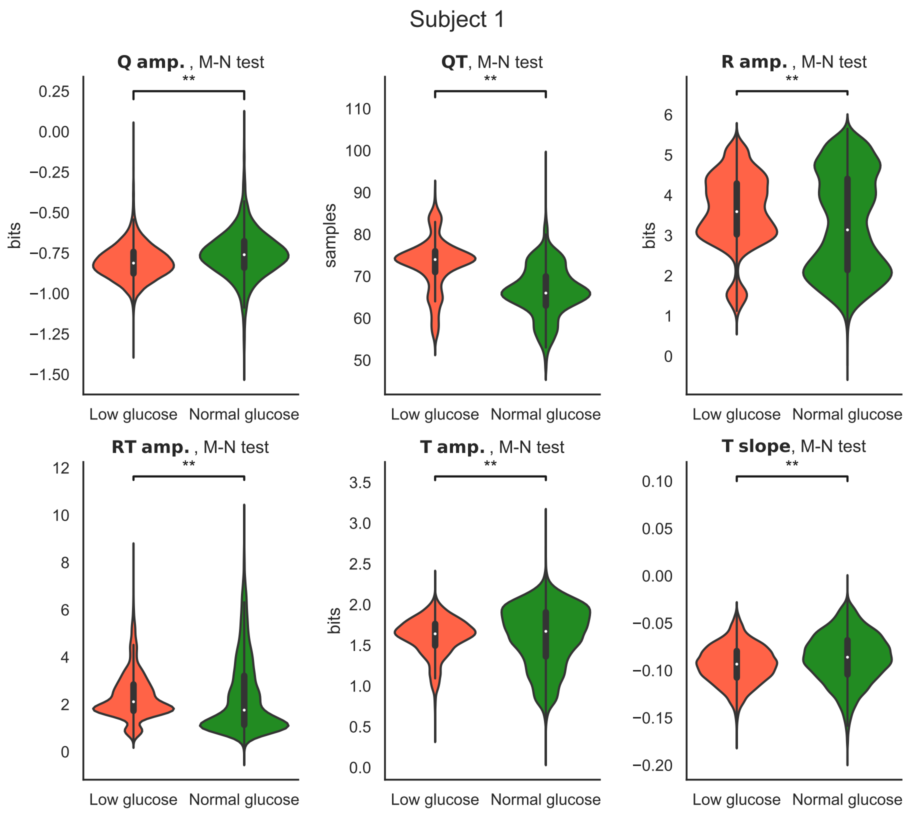
Subject 1
Q amp. , M-N test
**
bits
Low glucose
Normal glucose
QT, M-N test
**
samples
R amp. , M-N test
**
RT amp. , M-N test
**
T amp. , M-N test
**
T slope, M-N test
**

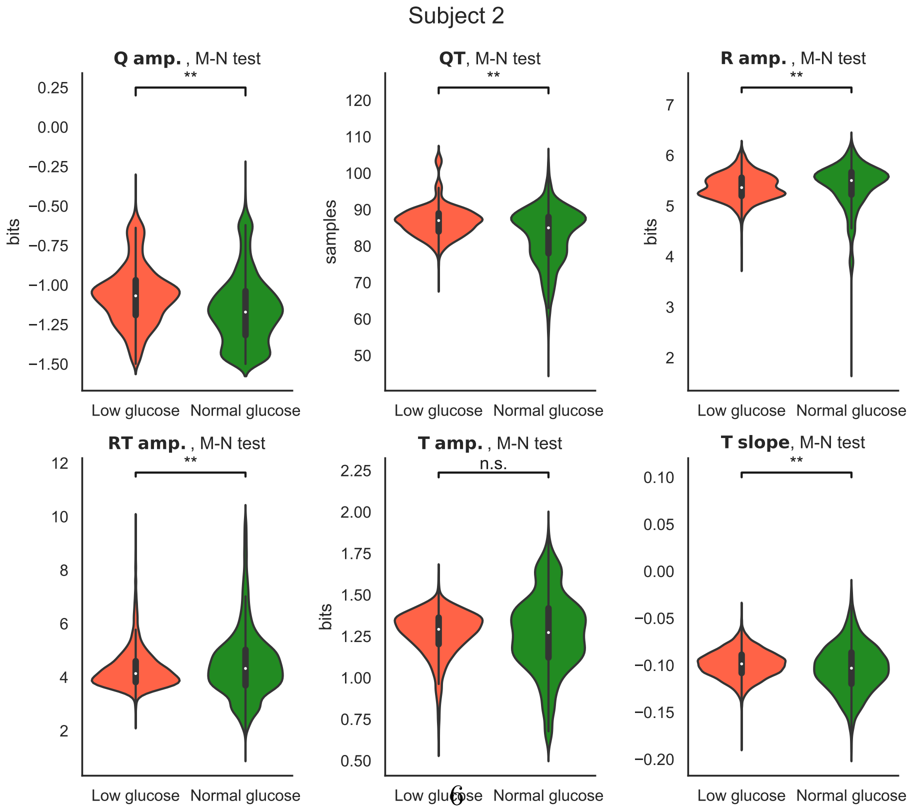
Subject 2
Q amp. , M-N test
**
bits
Low glucose
Normal glucose
QT, M-N test
**
samples
R amp. , M-N test
**
RT amp. , M-N test
**
T amp. , M-N test
n.s.
T slope, M-N test
**

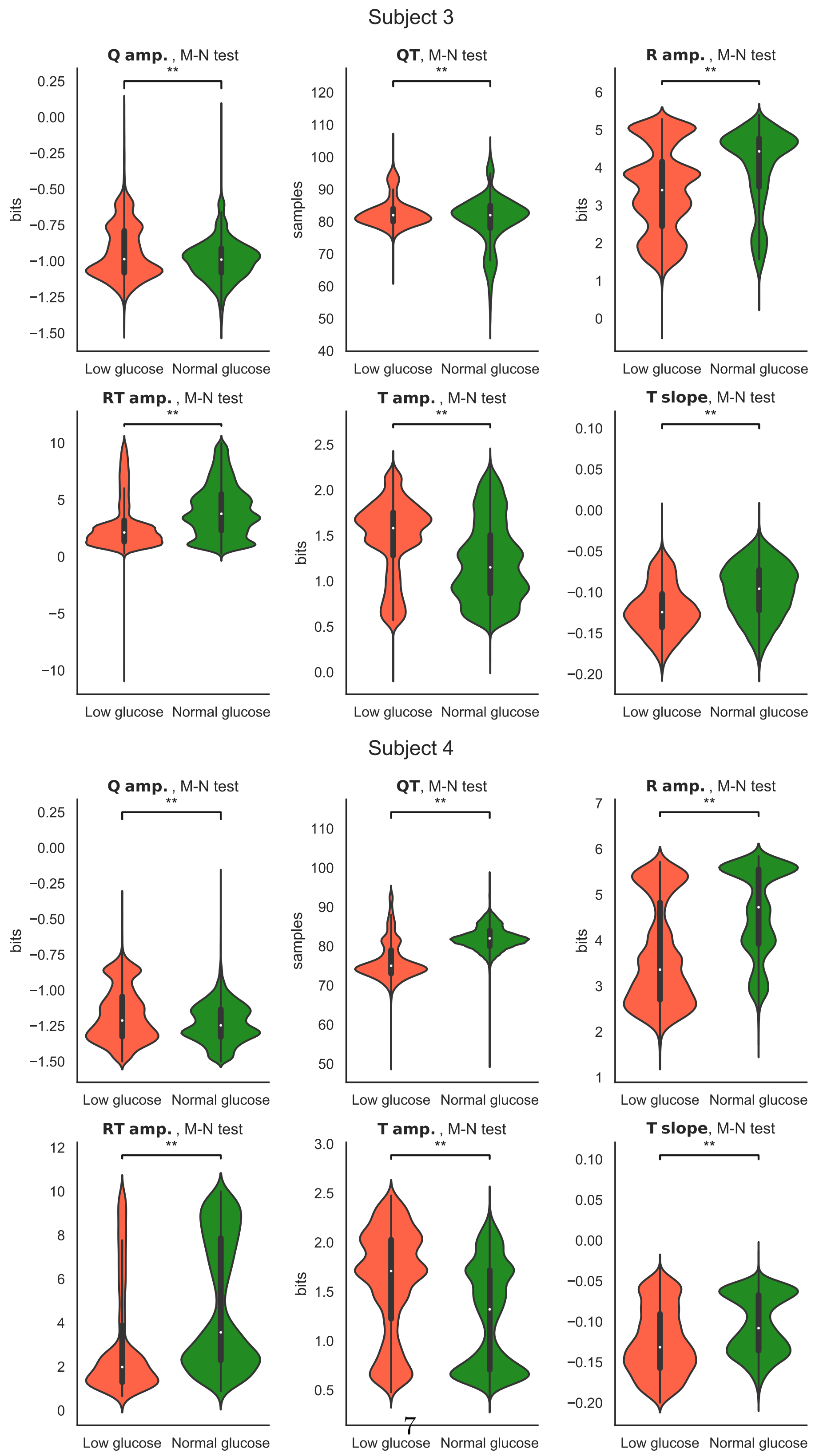




Figure 3: Mann-Whitney rank test on the extracted ECG parameters for each subject, figures a-d. All the statistical tests showed significant differences between the groups (low vs normal glucose level)
